# P-474. Effects of Antiretroviral Resistance on Outcomes and Healthcare Resource Use of People with HIV in the United States and Europe – A Real-World Survey

**DOI:** 10.1093/ofid/ofae631.673

**Published:** 2025-01-29

**Authors:** Mary J Christoph, Megan Chen, Seojin Park, Woodie Zachry, Cassidy Trom, Will Ambler, Oliver-Thomas Carter, Fritha Hennessy, Hannah Jones, Tim Holbrook

**Affiliations:** Gilead Sciences, Inc., Foster City, California; Gilead Sciences, Inc., Foster City, California; Gilead Sciences, Inc., Foster City, California; Gilead Sciences Inc, Foster City, California; Gilead Sciences, Inc., Foster City, California; Adelphi Real World, Bollington, England, United Kingdom; Adelphi Real World, Bollington, England, United Kingdom; Adelphi Real World, Bollington, United Kingdom, Bollington, England, United Kingdom; Adelphi Real World, Bollington, England, United Kingdom; Adelphi Real World, Bollington, United Kingdom, Bollington, England, United Kingdom

## Abstract

**Background:**

Resistance to key classes of ART leads to decreased treatment efficacy, virologic failure, and poorer clinical outcomes. Adding to current literature, this study reports current real-world trends between ART resistance mutations, healthcare resource use (HCRU), and people with HIV (PWH) outcomes.

**Figure:**
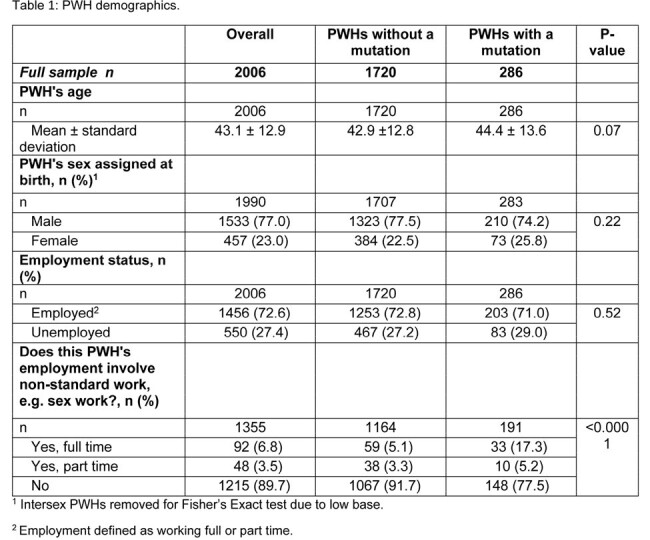

**Methods:**

Data were drawn from the Adelphi HIV Disease Specific Programme™, a real-world, retrospective, longitudinal survey from physicians and PWH in United States and Europe between July 2021-June 2023. Physicians provided medical chart data for PWH, including demographics, clinical characteristics, adherence, and hospitalization. Data were analyzed using bivariate comparisons, linear regressions, and negative binomial regressions.

**Figure:**
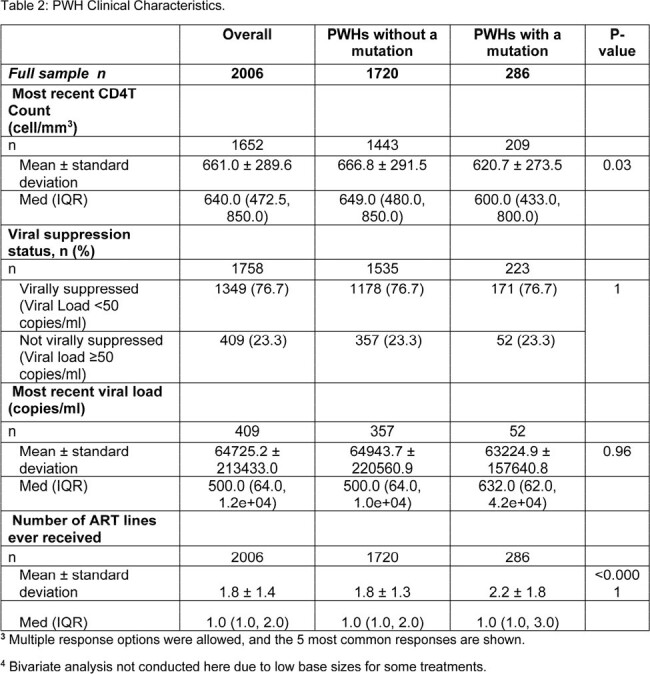

**Results:**

Physicians provided data for 2006 PWH. 14.3% of PWH had at least one mutation conferring resistance to an ART. PWH with a mutation (mPWH) received significantly more lines of ART than those without (nPWH) (mean ± SD: 2.2 ± 1.8 lines vs 1.8 ± 1.3 lines, respectively, p < 0.0001). More mPWH than nPWH had at least one side effect reported by the PWH themselves, (45.3% vs 39.2%, respectively, p = 0.058) and observed by their physician (35.1% vs 24.1%, respectively, p < 0.0001). Physicians reported fewer mPWH were considered to be “completely adherent” to their ART than nPWH (48.4% and 77.1% respectively, p < 0.0001).

In total, 9.6% of all PWH had at least one HIV-related hospitalization in the past 12 months. Significantly more mPWH had at least one HIV-related hospitalization in the past 12 months than nPWH (18.0% vs 8.4% p < 0.0001); and among those, mPWH had a higher mean number of visits than nPWH (mean ± SD: 1.7 ± 1.3 vs1.3 ± 0.9, respectively p = 0.02). Having at least one ART resistance mutation doubled the risk of hospitalization in the 12 months prior to data collection (incidence rate ratio [95% confidence interval]: 1/2.04 [1.34, 3.10], p = 0.001).

**Figure:**
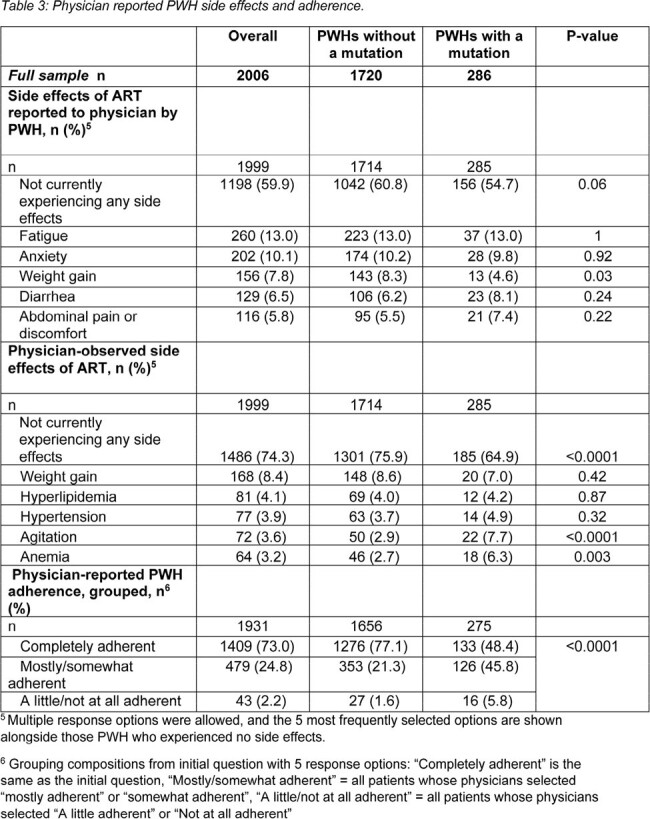

**Conclusion:**

In our secondary analysis of a real-world PWH database, mPWH had increased risk of hospitalization and side effects, and received more lines of ART, according to physicians. These findings show that ART resistance is associated with both higher HCRU and poorer PWH outcomes. Despite improvements in modern ARTs, treatment resistance still represents a significant burden on PWH and their healthcare systems.

**Figure:**
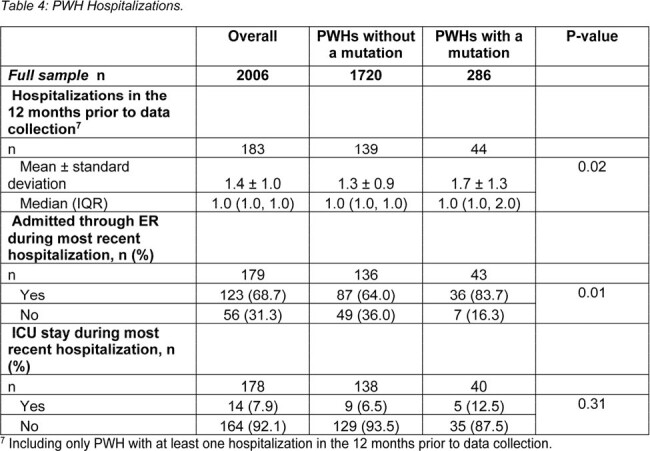

**Disclosures:**

**Mary J. Christoph, PhD, MPH**, AstraZeneca: Advisor/Consultant|AstraZeneca: Former employee|AstraZeneca: Stocks/Bonds (Private Company)|Gilead Sciences, Inc.: Employee|Gilead Sciences, Inc.: Stocks/Bonds (Private Company) **Megan Chen, MSPH**, Gilead Sciences, Inc.: Honoraria|Gilead Sciences, Inc.: Employee|Gilead Sciences, Inc.: Stocks/Bonds (Private Company) **Seojin Park, PharmD, MS**, Gilead Sciences, Inc.: Employee|Gilead Sciences, Inc.: Stocks/Bonds (Public Company) **Woodie Zachry, RPh, PhD**, Gilead Sciences, Inc.: Employee|Gilead Sciences, Inc.: Stocks/Bonds (Public Company) **Cassidy Trom, PharmD, AAHIVE**, Gilead Sciences, Inc.: Employee|Gilead Sciences, Inc.: Stocks/Bonds (Private Company) **Will Ambler, PhD**, Adelphi Real World: Employee|ViiV Healthcare: The Adelphi Real World Disease Specific Programme is wholly owned by Adelphi Real World; ViiV healthcare is one subscriber and paid for the analysis **Oliver-Thomas Carter, BSc**, Adelphi Real World: Employee|ViiV Healthcare: The Adelphi Real World Disease Specific Programme is wholly owned by Adelphi Real World; ViiV healthcare is one subscriber and paid for the analysis **Fritha Hennessy, PhD**, Adelphi Real World: Employee|ViiV Healthcare: The Adelphi Real World Disease Specific Programme is wholly owned by Adelphi Real World; ViiV healthcare is one subscriber and paid for the analysis **Hannah Jones, BSc, MRes**, Adelphi Real World: Employee **Tim Holbrook, BSc**, Adelphi Real World: Employee|ViiV Healthcare: The Adelphi Real World Disease Specific Programme is wholly owned by Adelphi Real World; ViiV healthcare is one subscriber and paid for the analysis

